# Supplementary material for: Ultrathin Photonic Polymer Gel Films Templated by Non-Close-Packed Monolayer Colloidal Crystals to Enhance Colorimetric Sensing
Source: Polymers (Basel). 2019 Mar 21;11(3):534. doi: 10.3390/polym11030534 (PMC6473593; doi:10.3390/polym11030534)
Supplement: Supplementary file 1 [file polymers-11-00534-s001.pdf]

Supporting information

Ultrathin Photonic Polymer Gel Films Templated by Non-Close-Packed Monolayer Colloidal Crystals to Enhance Colorimetric Sensing

Shimo Yu, Shun Dong, Xiuling Jiao, Cheng Li, and Dairong Chen**

National Engineering Research Center for Colloidal Materials, School of Chemistry and Chemical Engineering, Shandong University, 250100 Jinan, China.

*E-mail: chengli@sdu.edu.cn

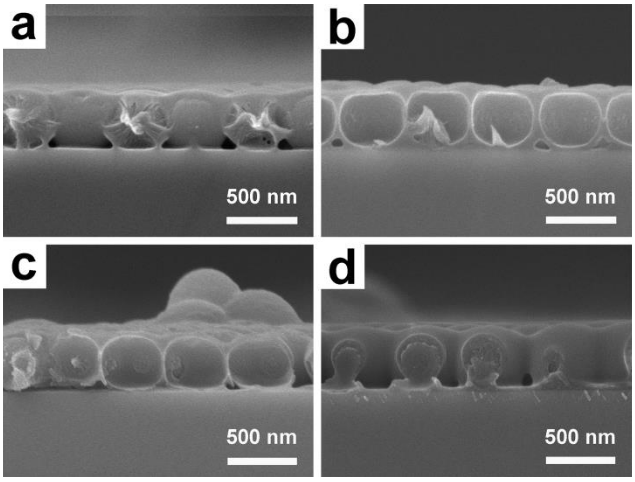

Figure S1. SEM images of UPPGF-0 after equilibration in pH 5.08 (a), pH 4.20 (b), pH 3.39 (c), and pH 2.57 (d) buffer solution.

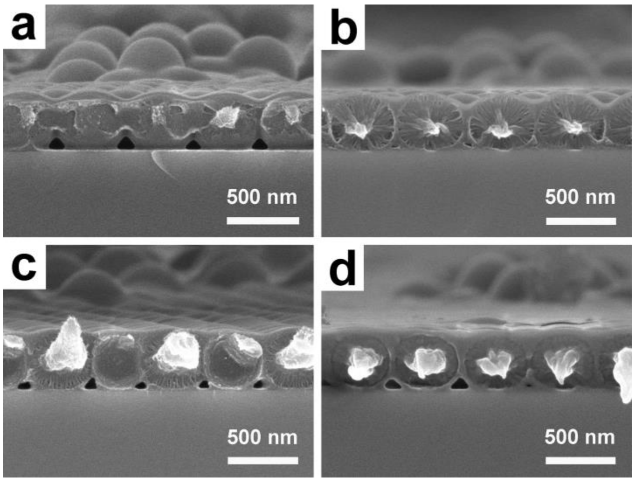

Figure S2. SEM images of UPPGF-2 after equilibration in pH 5.08 (a), pH 4.20 (b), pH 3.39 (c), and pH 2.57 (d) buffer solution.

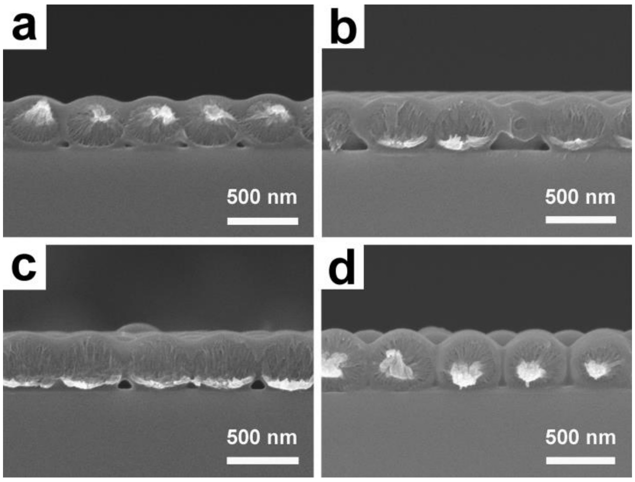

Figure S3. SEM images of UPPGF-6 after equilibration in pH 5.08 (a), pH 4.20 (b), pH 3.39 (c), and pH 2.57 (d) buffer solution.

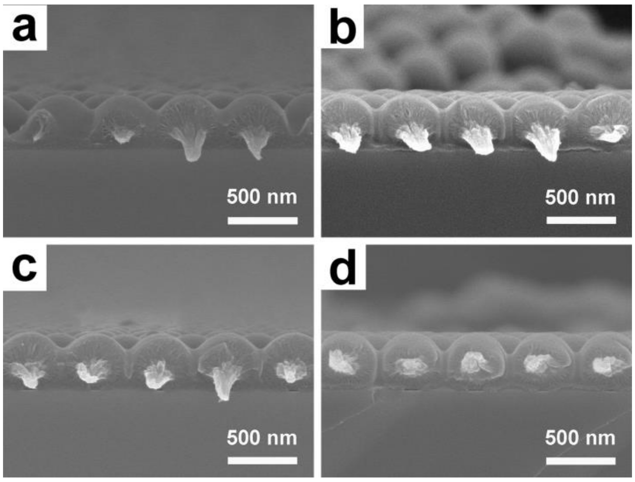

Figure S4. SEM images of UPPGF-8 after equilibration in pH 5.08 (a), pH 4.20 (b), pH 3.39 (c), and pH 2.57 (d) buffer solution.

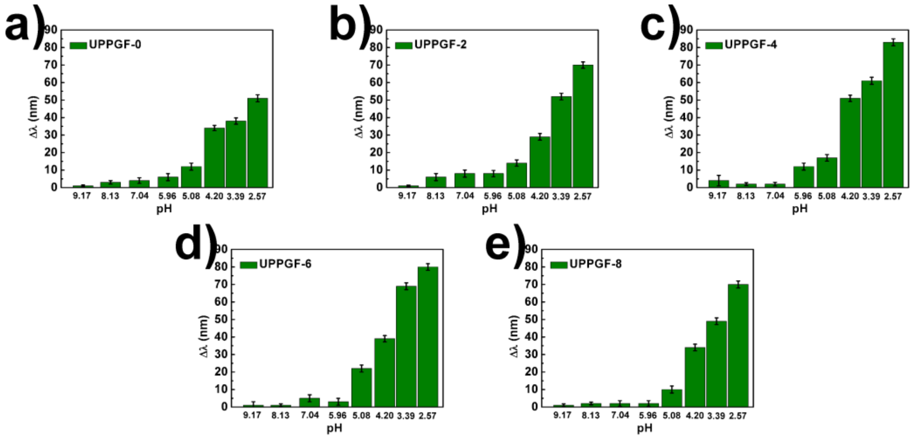

Figure S5. Corresponding shifts of the reflectance peak of UPPGF-x (x = (a) 0 min, (b) 2 min, (c) 4 min, (d) 6 min, (e) 8 min) after equilibration in pH buffer solution.
